# Phosphoric acid pretreatment of poplar to optimize fermentable sugars production based on orthogonal experimental design

**DOI:** 10.3389/fchem.2023.1119215

**Published:** 2023-02-22

**Authors:** Deming Chen, Wenjing Tang, Hui Wang, Yequan Sheng, Xin Tan, Yang Shi, Wei Fan, Shengbo Ge

**Affiliations:** ^1^ Ministry of Forestry Bioethanol Research Center, College of Materials Science and Engineering, Central South University of Forestry and Technology, Changsha, Hunan, China; ^2^ College of Biological and Food Engineering, Anhui Polytechnic University, Wuhu, Anhui, China; ^3^ Jiangsu Co-Innovation Center of Efficient Processing and Utilization of Forest Resources, International Innovation Center for Forest Chemicals and Materials, College of Materials Science and Engineering, Nanjing Forestry University, Nanjing, Jiangsu, China; ^4^ Key Laboratory of Functional Textile Material and Product of Ministry of Education, School of Textile Science and Engineering, Xi’an Polytechnic University, Xi’an, Shanxi, China

**Keywords:** enzymatic hydrolysis, orthogonal experiment, poplar, phosphoric acid pretreatment, bio-energy

## Abstract

The recalcitrant structure of raw poplar limited the production of fermentable sugars when applied as the material in the pretreatment of biochemical conversions. Phosphoric acid pretreatment is an efficient method to destroy the compact lignocellulose matrix presence in the poplar. In this study, phosphoric acid pretreatment of poplar was optimised by an orthogonal experimental design [L_9_(3^3^)] to improve enzymatic digestibility through investigating the effects of reaction temperature, time duration, and phosphoric acid concentration. The optimal conditions were selected based on the variance of chemical compositions, hemicellulose removal ratio, and delignification of the woody material after pretreatment. The optimum enzymatic hydrolysis yield of up to 73.44% was obtained when the phosphoric acid pretreatment performed at 190°C for 150 min under 1.5% of v/v phosphoric acid concentration.

## Highlights

• Phosphoric acid (H_3_PO_4_) was used for the pretreatment of poplar.
• Orthogonal experimental design was used to optimize pretreatment conditions.
• Phosphoric acid efficiently removed hemicellulose, resulting in more glucose yield.
• Reaction temperature and H3PO4 concentration were two important factors.

## 1 Introduction

The use of alternative transportation fuels, such as biofuels, to reduce greenhouse gas emissions has grown in recent years (Patel and Shah, 2021; Zhang et al., 2022). Among the various biofuels, the lignocellulosic biofuel industry has seen a significant increase in its growth (Huang et al., 2018; Wang et al., 2022a). Due to their saccharide-rich nature, abundance, and low cost, lignocellulosic materials are considered to be cost-effective and would not create an energy-food conflict (Zhang et al., 2021; Wang et al., 2022b). In view of its prevalence as one of the most frequent fast-growing woods in the world, poplar is regarded as a promising material for the production of biofuels (Ying et al., 2021). For lignocellulosic biomass to be converted into biofuel, a bio-chemical conversion process that is economically and efficiently designed is necessary (Orejuela-Escobar et al., 2021; Wu et al., 2023). A typical biofuel production process includes a pretreatment, enzymatic hydrolysis, and fermentation (Hoang et al., 2021). The chemical value of the woody materials (e.g., poplar) could be fully utilized by proper pretreatment; therefore, it is crucial that the pretreatment process is efficient in order to ensure a high level of production output (Kumari and Singh, 2018).

Pretreatment represents one of the biochemical processes to increase the accessibility of the saccharides in the lignocellulosic material like poplar by releasing the recalcitrance of the woody material (Mankar et al., 2021). To release the sugars and render the biomass more susceptible to cellulases, a variety of pretreatment methods have been developed, including hot water, dilute acid, steam, and organosolv (Rezania et al., 2020; Liu et al., 2021; Nnaemeka et al., 2021). Pretreatment with diluted acid is one of the most widely used pretreatment methods for increasing the digestibility of biomass by removing hemicellulose efficiently (Dien et al., 2006). In comparison with other widely used pretreatment methods like alkali pretreatment, dilute acid pretreatment has received wide attention due to its lower capital and operating costs (Saville et al., 2016), low enzymes requirement (Bensah and Mensah, 2013), and improved cellulose accessibility and digestibility (Baadhe et al., 2014). There is, however, a serious limitation in the development of acid pretreatment processes due to the potential toxicity and equipment corrosion associated with strong acids such as sulfuric acid and hydrochloride acid (Roy et al., 2020). As an alternative to common acid pretreatment methods, phosphoric acid pretreatment provides lower activity and toxicity. Due to its further use of pretreatment waste as a fertilizer and neutralizer, phosphoric acid has been increasingly involved in the dilute acid pretreatment process (Yu et al., 2019). It has been demonstrated that different concentrations of phosphoric acid in the pretreatment are effective on a variety of agricultural residues, grasses, eucalyptus, and poplar types of wood (Dey et al., 2021; Zeng et al., 2021; Tong et al., 2022). In order to optimize the condition of pretreatment and enzymatic hydrolysis, several different optimization strategies have been conducted (Ji et al., 2021; Hou et al., 2022). Different phosphoric acid concentration (70%, 75%, and 80%) was employed for the pretreatment of two types of weed biomass by enzymatic hydrolysis (Siripong et al., 2016). The optimal phosphoric acid concentration for glucose yield was 80% and 75% for *A. aspera* and *S. acuta*, respectively. Kim and Mazza (2008) employed a central composite design (CCD) and reported that the maximum digestibility of pretreated flax shives was 94.8% under 86.2% phosphoric acid concentration, 110.5 min and 13.1 FPU/g cellulose at 50°C and 120 h. Additionally, 75% phosphoric acid was used to treat bark and core fibers, which led to enhanced enzymatic hydrolysis (Premjet et al., 2022). Tong et al. (2022) introduced one surfactant agent JFC into the dilute phosphoric acid plus steam explosion pretreatment of poplar with maximum enzymatic saccharification rate of 84.62%. These studies indicated that phosphoric acid pretreatment is generally done with higher phosphoric acid concentrations or combined with other pretreatment methods. Moreover, very few studies have been conducted in which phosphoric acid was pretreated in a ball mill system. Therefore, it is possible that optimizing the conditions of phosphoric acid pretreatment in the ball mill system could be vital to the development of industrial applications for phosphoric acid pretreatment in the future.

The orthogonal design of experiments is an effective means of optimizing new experimental methods. Multiple factors with different levels of involvement in the reaction can be optimized through reasonable numbers of experiments (Ji et al., 2014). The glucose yield in the subsequent enzymatic hydrolysis could be an efficient indicator of the pretreatment efficiency (Yao et al., 2020). Therefore, the purpose of this study was to apply an orthogonal design for the phosphoric acid pretreatment in the ball mill pretreatment system in order to optimize the enzymatic hydrolysis yield. The chemical compositions, hemicellulose removal ratio, and delignification of pretreated materials were also investigated to elucidate the reason behind the high enzyme digestibility of the optimal pretreatment condition. Finally, the confirmatory experiment is conducted with the optimised factors.

## 2 Materials and methods

### 2.1 Materials and equipment

The raw material of poplar wood powder (20–80 mesh) was purchased from Jiangsu-Zhangjiagang imported timber trading market. Phosphoric acid and sodium citrate (Analytical Reagent, AR) were obtained from Shanghai Reagent Co., Ltd. The enzymatic hydrolysis was assisted by Cellulase Cellic@CTec2 (Sigma Aldrich-Shanghai). The filter paper activity of commercial cellulose was 200.0 FPU/mL. Deionised water (AR) from Nanjing Danuo Chemical Industry Co., Ltd. was used in solution preparation and chemical reaction. Pretreatment of poplar powder was performed in the ball mill (BGP-R8) bought from Hefei Kejing Material Technology Co., Ltd. Temperature of acid hydrolysis in chemical composition analysis was controlled by water bath (HWS-24) from Shanghai Hengyi Scientific Instrument Co., Ltd. Diluted acid hydrolysis of hydrolysed wood powder was autoclaved in pressurised heating steriliser (XFH-30MA, Zhejiang Xinfeng Medical Equipment Co., Ltd.). Hydrolysate and the solid residue were separated by the multi-purpose water pump (SHZ-D, Shanghai Lichenbang Instrument Technology Co., Ltd.) after the reaction. The composition of ash and the solid residue was calculated by combustion in the muffle furnace (KSL-1200X) manufactured from Hefei Kejing Material Technology Co., Ltd. Based on the analysis of the chemical composition of woody material, enzymatic hydrolysis of pretreated wood was conducted in the shaking incubator (MQT-60NR Shanghai Minquan Instrument Co., Ltd.). The concentration of hydrolysed sugars was analysed by high-performance liquid chromatography (HPLC, E2695, Waters Corporation of America) with HPX-87H column (Aminex, Bio-Rad) and RID detector.

### 2.2 Orthogonal experimental design of pretreatment conditions

Poplar wood powder was pretreated in the ball mill. In each ball milling vessel, 4 g of poplar wood powder was added and immersed in various concentrations of 0.5%, 1%, and 1.5% v/v phosphoric acid with a solid-to-liquid ratio of 1:7. Five of 4 mm grinding balls together with 5 of 8 mm grinding balls (ten grinding balls in total) were used in the pretreatment to ensure the mixing efficiency. The reaction was conducted at different pretreatment temperatures (170°C, 180°C, and 190°C) and different pretreatment duration (120, 150, and 180 min) with a fixed grinding speed of 300 rpm/min. The orthogonal experimental design is shown in Table 1. A vacuum was applied to separate the liquid from the solid after pretreatment. Next, solid materials were rinsed to neutrality with water and then placed in a refrigerator at 4°C for further application.

**TABLE 1:** The factors and levels of the pretreatment conditions.

Levels	Factors		
	A	B	C
	Reaction temperature (°C)	Reaction time (min)	Phosphoric acid concentration (%, v/v)
1	170	120	0.5
2	180	150	1
3	190	180	1.5

The range analysis is used to determine the optimal level of each factor and assess the significance level of all factors. In the range analysis, the K value and R value are calculated. The K value of each level of a factor is the sum of three values with the same level. The R value for each factor is the difference between the maximum and minimal K values of the three levels. The influence of one factor on the value would be the most significant if it has the largest R value (Zhang et al., 2019).

### 2.3 Chemical composition of materials

Compositional analysis of the ball-milled poplar was performed according to the NREL protocol (Sluiter et al., 2008). The concentrated sulfuric acid was first applied to hydrolyse the pretreated materials spread at the bottom of the hydrolysis bottle. The initial hydrolysis was conducted in a 30°C water bath for 1 h with manual stirring every 10 min for high reaction efficiency. Then, further hydrolysis of the material using diluted acid was performed in the autoclave at 121°C for 1 h. After the filtrate was separated from the hydrolyzate, acid-soluble lignin (analysed *via* UV-vis at 205 nm) and sugar (analysied *via* HPLC) were determined from the filtrate. Insoluble solid was collected when it was washed back to neutral. The dry weight of the crucible weighed out the insoluble components after being burned in a muffle furnace (525°C).

### 2.4 Enzymatic hydrolysis

The hydrolysis was set up in a 150 mL conical flask with a lid containing 30 mL reaction system (Sheng et al., 2021a). The enzymatic hydrolysis was conducted with 1% (w/v) cellulose content and the cellulase of 20 FPIU/g cellulose followed by shaking in an incubator at 50°C for 72 h with a speed of 150 rpm. The pH of the reaction system was maintained at 4.8 with sodium citrate buffer. Glucose yield was calculated using the glucose released in the supernatant divided by the theoretical glucan content in pretreated poplar. The data were recorded based on results of two replicates.

### 2.5 Calculations

The hemicellulose removal ratio, the degree of delignification, and enzymatic digestibility were calculated according to the following equations:

$$Hemicellulose\;removal\;yield\;\left(\%\right)=1-\frac{hemicellulose\;in\;pretreated\;poplar\;residues\;\left(g\right)}{hemicellulose\;in\;the\;raw\;poplar\;\left(g\right)}\times 100\%$$

$$Delignification\;\left(\%\right)=1-\frac{acid\;insoluble\;lignin\;in\;pretreated\;poplar\;residues\;\left(g\right)}{acid\;insoluble\;lignin\;in\;the\;raw\;poplar\;\left(g\right)}\times 100\%$$

$$Enzymatic\;digestibility\;\left(\%\right)=\frac{glucose\;in\;enzymatic\;hydrolysate\;\left(g\right)}{initial\;glucose\;in\;substrate\;\left(g\right)}\times 100\%$$

## 3 Results and discussion

### 3.1 Effect of different factors/levels on substrates digestibility

The experiments are designed to optimise the main factors in the phosphoric acid pretreatment process of poplar. Orthogonal experimental design has been widely used to optimise pretreatment conditions on lignocellulosic biomass (Xu et al., 2011). The orthogonal experiments were conducted in order to maximise glucose yield by controlling reaction temperature (A), reaction time (B), and phosphoric acid concentration (C) in the ball mill pretreatment. These factors were chosen because they are important factors that influences on enzymatic hydrolysis (Zhou and Xu, 2019). Based on this experimental design, poplar was pretreated at different temperatures (170°C–190°C) and different reaction times (120–180 min) with 4.0 g of poplar and 28 mL phosphoric acid solutions with different phosphoric acid concentrations in this study. Values of independent process variables were studied and the results of enzymatic hydrolysis obtained from nine different combinations of reaction conditions are shown in Table 2.

**TABLE 2:** Results of glucose yields of all pretreated substrates.

	Reaction temperature (°C)	Reaction time (min)	Phosphoric acid concentration (%, v/v)	Enzymatic digestibility^a^ (%)
PA1	170	120	0.5	26.64
PA2	170	150	1	31.45
PA3	170	180	1.5	39.27
PA4	180	120	1	39.96
PA5	180	150	1.5	54.66
PA6	180	180	0.5	32.14
PA7	190	120	1.5	67.44
PA8	190	150	0.5	47.94
PA9	190	180	1	58.75
Range R	25.59	1.29	18.22	—
Order	1	3	2	

^a^ Enzymatic digestibility was calculated based on the glucose released from pretreated poplar after 72 h of saccharification using cellulase.

**FIGURE 1:**
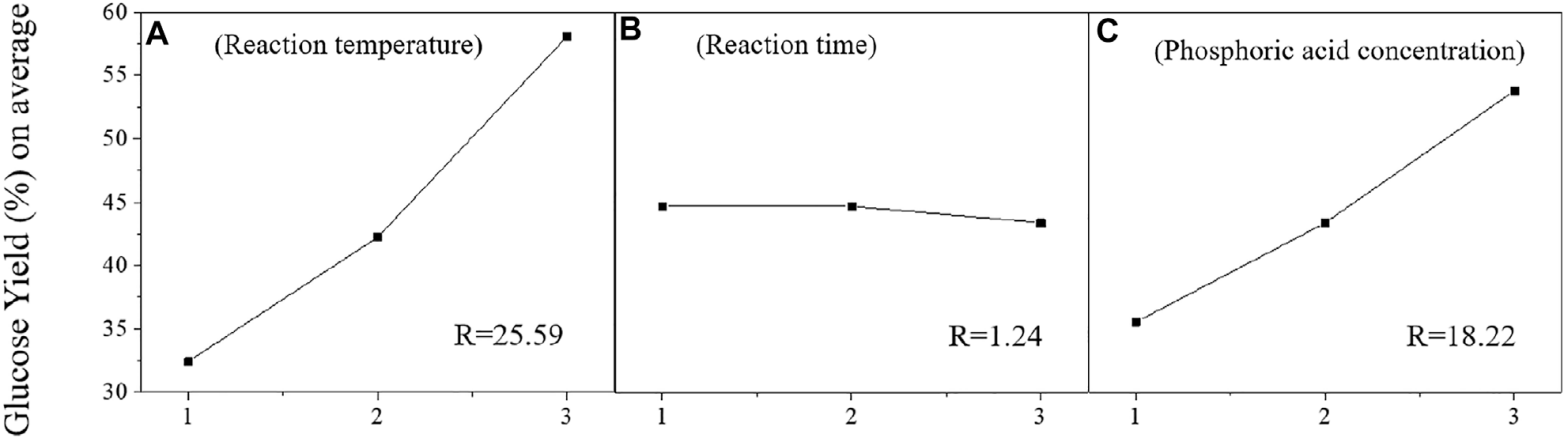
Analysis of orthogonal experiment factors on glucose yield [reaction temperature **(A)**, reaction time **(B)**, and phosphoric acid concentration **(C)**].

Figure 1 shows the effects of three factors, including reaction temperature (A, level 1: 170°C, level 2: 180°C, level 3: 190°C), reaction time (B, level 1: 120 min, level 2: 150 min, level 3: 180 min), and phosphoric acid concentration (C, level 1: 0.5 v/v%, level 2: 1.0%, level 3: 1.5%), on glucose yield of pretreated materials. The results showed that the glucose yield of pretreated poplar was significantly increased when higher reaction temperature and phosphoric acid concentration were applied (Figure 1). Specifically, the glucose yield was enhanced from 32.45% and 35.57% to 58.04% and 53.79%, with the improvement of reaction temperature (from 170°C to 190°C) and phosphoric acid concentration (from 0.5% to 1.5%), respectively. A similar observation was reported on sulfuric acid hydrolysis of rice hulls, in which reaction temperature was found as the most important parameter to increase the hydrolysis yield (Wei et al., 2009).

Interestingly, when the reaction time increased from 120 to 150 min and even 180 min, the glucose yield on average, show a slight decrease from 44.68% to 43.39%. This indicates that the effect of reaction time (in the range of 120–180 min, which is the most common time duration of pretreatment methods) on enzymatic hydrolysis of pretreated substrates is insignificant when compared with the effects of reaction temperature and phosphoric acid concentration. Extreme difference (Range, R) shows how much the dependent variable fluctuates when the factors change. According to the calculation of the R-value of each factor (Figure 1), it can be found that the influence of reaction time on the glucose yield was the lowest (1.24) compared to reaction temperature (25.59) and phosphoric acid concentration (18.22). Thus, both reaction temperature and phosphoric acid concentration strongly influence the digestibility of phosphoric acid-pretreated materials, while the effect of reaction time was negligible. As shown from the orthogonal experiment results (Figure 1; Table 2), the optimal reaction conditions were 190°C of reaction temperature, 150 min reaction time, and 1.5% v/v of phosphoric acid concentration.

The analysis of variance (ANOVA, including the degree of freedom (f), sums of squares (s), variance (V) and the regression coefficients (including *F*-value and *p*-value) of the designed model were shown in Table 3 to describe the relationship between the three factors and the glucose yield of the corresponding pretreated material in the enzymatic hydrolysis. A high *F*-value of around 20 suggests a strong correlation between factors and their dependents (Kim et al., 2011). The high *F*-value of reaction temperature and phosphoric acid were 34.34 and 17.20 (Table 3), respectively, indicating that these two factors strongly correlate to the enzymatic hydrolysis yield of the corresponding pretreated materials. Moreover, the *p*-value is also an important coefficient for understanding the mutual interaction pattern between the variables. As shown from the results, the *p*-value of reaction temperature (0.03) was below 0.05, suggesting this factor was statistically significant to the changes of digestibility of the pretreated materials. Therefore, reaction temperature and phosphoric acid concentration in the ball mill pretreatment are highly influential to the digestibility of the pretreated poplar.

**TABLE 3:** Variance analysis results for all parameters.

Factors	Degrees of freedom (f)	Sum of squares (s)	Variance (V)	F value	*p*-value
Reaction temperature (°C)	2	1000.21	500.11	34.34	0.03
Reaction time (min)	2	3.35	1.68	0.12	0.90
Phosphoric acid concentration (%, v/v)	2	501.12	250.56	17.20	0.05

### 3.2 Effect of phosphoric acid pretreatment on the chemical composition of poplar

Based on the orthogonal design shown in Table 4, the chemical compositions of pretreated poplar with different factors and levels were compare. Phosphoric acid pretreatment significantly decreased hemicellulose content from 16.1% (raw poplar) to 3.06%–11.3%. The highest hemicellulose removal ratio was up to 83.8%. This suggested that phosphoric acid can effectively remove hemicelluloses, making cellulose easier to be attacked by enzyme (Dien et al., 2006). Similarly, xylose is considered as one of the inhibitors for the following enzymatic digestion (Castro et al., 2014). Xylose was also released (16.1% to 3.06%–9.33% after pretreatment) during phosphoric acid pretreatment. The results showed the feasibility of phosphoric acid in the pretreatment.

**TABLE 4:** Chemical compositions of phosphoric acid pretreated biomass.

Biomass	Glucan (%)	Xylan	Acid soluble lignin	Acid insoluble lignin	Hemicellulose removal ratio	Delignification^a^
Raw material	44.83 ± 1.25	16.07 ± 0.15	2.05 ± 0.06	24.24 ± 0.58		
PA1	41.31 ± 1.62	9.33 ± 0.42	2.07 ± 0.03	18.97 ± 0.05	40.05	24.04
PA2	39.75 ± 0.16	7.30 ± 0.04	1.68 ± 0.01	19.15 ± 0.63	61.38	23.57
PA3	42.02 ± 0.30	5.16 ± 0.02	1.37 ± 0.05	20.48 ± 0.19	72.70	21.12
PA4	42.64 ± 0.57	5.49 ± 0.08	1.41 ± 0.05	20.34 ± 0.42	70.95	21.48
PA5	41.50 ± 0.36	3.78 ± 0.04	1.27 ± 0.03	21.19 ± 0.45	80.00	18.92
PA6	40.54 ± 0.85	5.61 ± 0.01	1.42 ± 0.02	21.05 ± 0.37	70.32	18.88
PA7	42.26 ± 0.31	3.16 ± 0.09	1.16 ± 0.02	23.22 ± 0.14	83.28	11.99
PA8	39.35 ± 0.10	3.99 ± 0.04	1.36 ± 0.06	21.38 ± 0.64	78.89	17.90
PA9	40.83 ± 0.23	3.06 ± 0.07	1.16 ± 0.03	24.25 ± 0.35	83.81	8.27

^a^ Delignification was calculated by the mass balance analysis based on acid insoluble lignin content.

**FIGURE 2:**
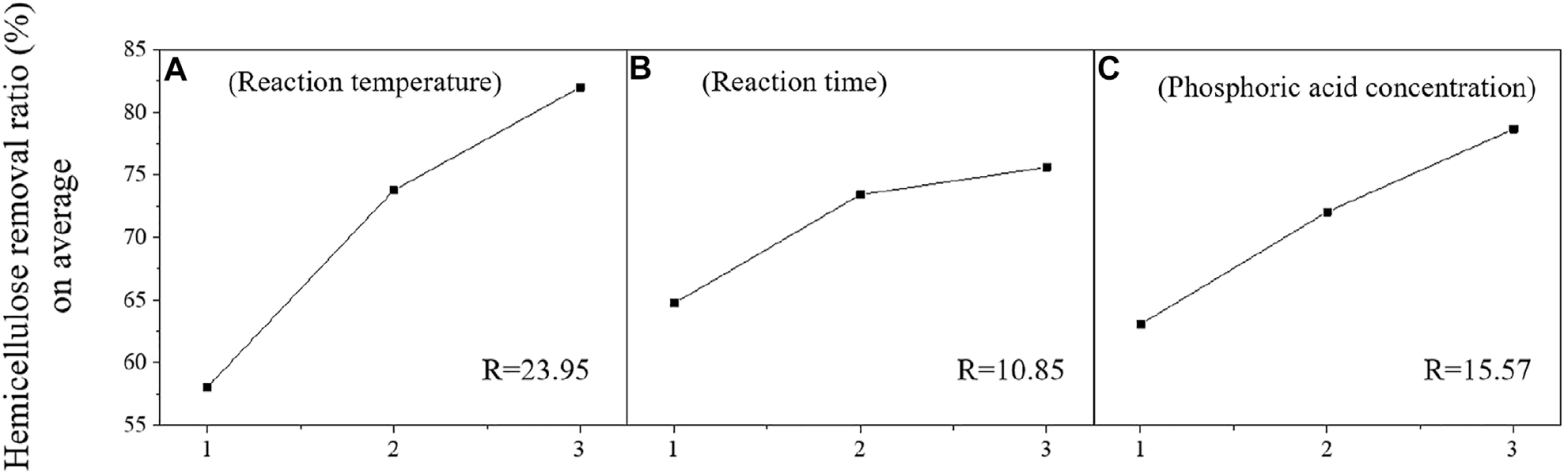
Analysis of orthogonal experiment factors on hemicellulose removal ratio [reaction temperature **(A)**, reaction time **(B)**, and phosphoric acid concentration **(C)**].

The effects of three factors including reaction temperature, reaction time, and phosphoric acid concentration, on the hemicellulose removal ratio of pretreated materials were shown in Figure 2. The hemicellulose removal ratios, on average, were all increased with reaction temperature, reaction time, and phosphoric acid concentration (Figure 2). This indicated that any factors that influence the severity of pretreatment reaction conditions would affect the removal ratio of hemicellulose. As revealed from the R-value of each factor (Figure 2), the influence of reaction temperature on the hemicellulose removal ratio (23.95) was significantly greater than reaction time (10.85) and phosphoric acid concentration (15.57). This shows that the reaction temperature of pretreatment has a stronger influence than the phosphoric acid concentration and reaction time in hemicellulose removal.

**FIGURE 3:**
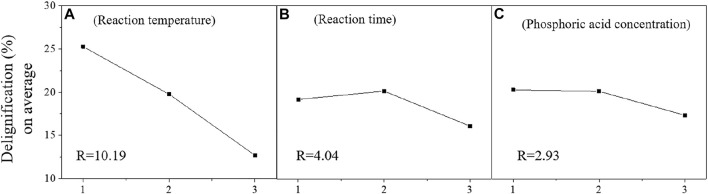
Analysis of orthogonal experiment factors on delignification [reaction temperature **(A)**, reaction time **(B)** and phosphoric acid concentration **(C)**].

Table 4 displays the delignification ratio ranged from 8.27% to 24.0% under different pretreatment conditions. The analysis between the three factors and the delignification ratio is shown in Figure 3. Interestingly, the delignification ratio decreased from 22.9% to 10.2% when the reaction temperature increased from 170°C to 190°C. The increment of acid-insoluble lignin contents in pretreated substrates could be related to the higher removal ratio of hemicellulose (Sheng et al., 2021b). However, the glucose yield on average was increased from 32.5% to 58.0% as the reaction temperature enhanced despite the lignin is usually considered a major inhibitor of enzymatic hydrolysis. Besides the changes in the total amount of sugars and lignins after pretreatment, the structural changes in lignins during phosphoric acid pretreatment and the physicochemical property of pretreated materials should also be considered (Pielhop et al., 2016). In short, some of the pretreated lignin could improve enzymatic hydrolysis (Lai et al., 2014).

The R-value between the three factors and the delignification ratio was also calculated (Figure 3). The effects of reaction time (4.04) and phosphoric acid concentration (2.93) were weaker than reaction temperature (10.19). To sum up, acid pretreatment hardly removes the lignins in the pretreated materials compared to hemicellulose removal ratio (Lai et al., 2018). Therefore, delignification ratio of acid pretreatment is not important as hemicellulose removal ratio. This might be the reason why the three factors show different trends when they are correlated to the delignification ratio (Figure 3).

### 3.3 The relationship between hemicellulose removal and the enzymatic digestibility of pretreated poplar

**FIGURE 4:**
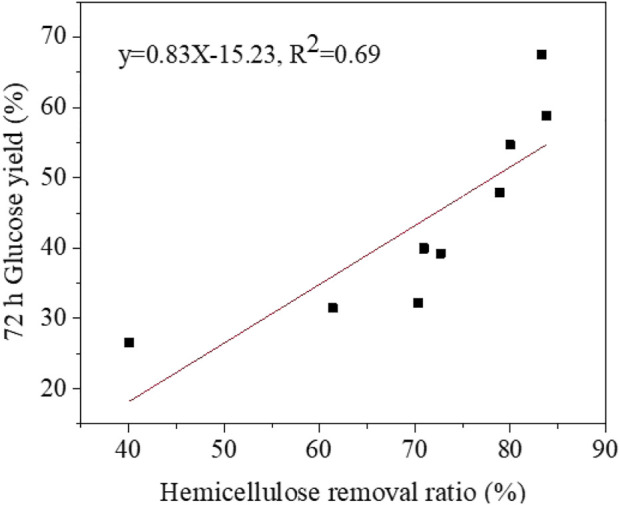
The relationship between hemicellulose removal and 72 h glucose yield of pretreated poplar.

The removal of xylan played an important role in the enzymatic hydrolysis of lignocellulosic biomass. Penttilä et al. (2013) analysed the limiting effect on enzymatic hydrolysis of cellulose by hydrolysing nanocellulose samples with different xylan content. The binding of xylan and nanocellulose limited the hydrolysis of crystalline cellulose because of the enhancement of cellulose crystallinity during hydrolysis. A regression curve was plotted based on the data of hemicellulose removal ratio versus 72 h glucose yield (Figure 4). The final glucose yield of pretreated poplar was positively correlated with hemicellulose removal ratio, and a slight relationship with the determination coefficient was observed (*R* ^2^ = 0.69). Wu et al. (2018) also found a slight correlation between xylan removal and enzymatic digestibility (*R* ^2^ = 0.70) by combining autohydrolysis pretreatment and alkaline post-extraction. The correlation between hemicellulose removal ratio and final enzymatic hydrolysis yield might be stronger within a certain range. For example, the 72-h glucose yield of PA7 (67.44%) was significantly higher than PA9 (58.75%). However, the hemicellulose removal ratio of PA7 (83.28%) and PA9 (83.81%) were very similar. The results showed that the positive effect of hemicellulose removal with phosphoric acid pretreatment on enzymatic digestibility was significant only at the hemicellulose removal ratio of below 75% (*R* ^2^ = 0.84). Moreover, some other physical and chemical properties of pretreated materials including lignin structure and cellulose accessibility, should be considered when the hemicellulose ratio is high and relatively close to each other (Lai et al., 2018; Sheng et al., 2021a).

### 3.4 Enzymatic hydrolysis under the optimal pretreatment condition

**FIGURE 5:**
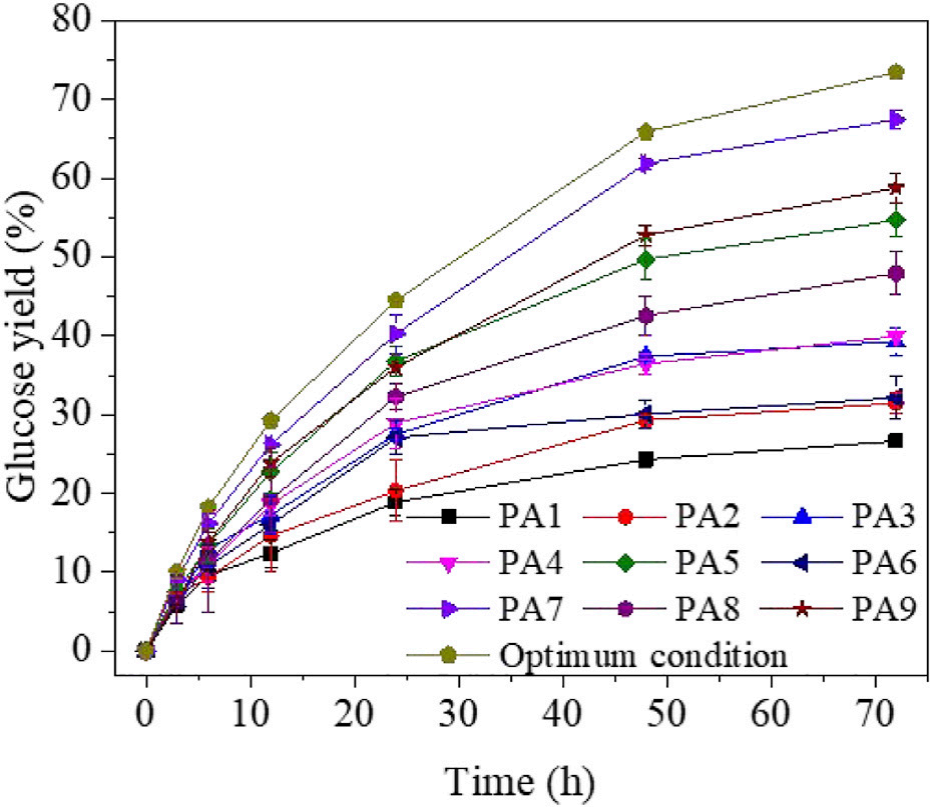
Time course of enzymatic hydrolysis of phosphoric acid pretreated substrates 1% cellulose (w/v).

Poplar was pretreated with the optimal condition (190°C, 150 min, and 1.5% v/v phosphoric acid concentration) to examine whether the optimal pretreatment condition can result in the highest enzymatic digestibility. Phosphoric acid pretreated poplar substrates were enzymatically hydrolysed with 20 FPU/g glucans of cellulase and 1% glucan (w/v) (Figure 5, condition PA10). As a result, it was observed that the 72-h glucose yield of pretreated poplar under optimum conditions increased to 73.44% which was up to three times higher than the yields before optimisation (e.g., PA1, 26.64%). Moreover, the initial hydrolysis rate of optimum condition (3.35 g/L/h) was higher than other groups. Therefore, with the assistance of orthogonal design, this optimisation process was a success.

## 4 Conclusion

Optimisation of phosphoric acid pretreatment of poplar was conducted by orthogonal experimental design. The optimal conditions to obtain the highest glucose yield were heating at 190°C for 150 min under 1.5% v/v of phosphoric acid. The final glucose yield of pretreated poplar under optimum conditions was 73.44%, significantly higher than the one before optimisation (26.64%). It was found that phosphoric acid pretreatment led to significant reductions in hemicellulose removal (removal ratio of up to 83.81%), and that 72-h glucose yield was positively correlated with hemicellulose removal. This study has therefore successfully demonstrated that phosphoric acid is a useful pretreatment agent for the degradation of recalcitrant poplar matrix, and after orthogonal optimization, enzymatic digestibility increased by 73.44%.

## Data Availability

The original contributions presented in the study are included in the article/Supplementary Material, further inquiries can be directed to the corresponding authors.
