# Supplementary figures and images for: Phosphoric acid pretreatment of poplar to optimize fermentable sugars production based on orthogonal experimental design
Source: Front Chem. 2023 Feb 22;11:1119215. doi: 10.3389/fchem.2023.1119215 (PMC9993246; doi:10.3389/fchem.2023.1119215)

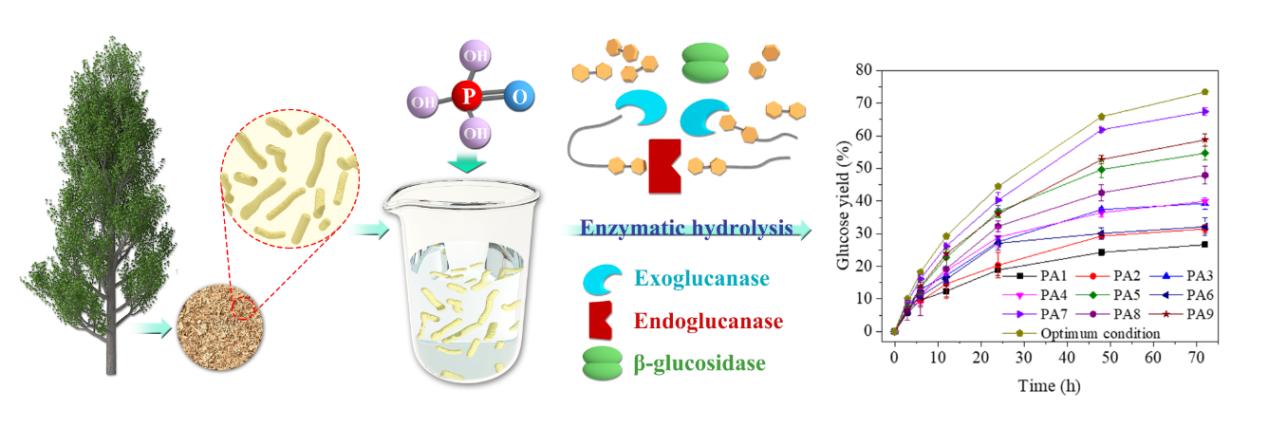

Supplement: Supplementary file 1 [file DataSheet1.docx]
